# Organoids as a new model system to study neural tube defects

**DOI:** 10.1096/fj.202002348R

**Published:** 2021-04

**Authors:** Yu Wu, Sisi Peng, Richard H. Finnell, Yufang Zheng

**Affiliations:** 1 Department of Cellular and Developmental Biology, School of life sciences, Fudan University, Shanghai, China; 2 Obstetrics & Gynecology Hospital, The institute of Obstetrics and Gynecology, Fudan University, Shanghai, China; 3 Center for Precision Environmental Health, Departments of Molecular and Cellular Biology, Molecular and Human Genetics and Medicine, Baylor College of Medicine, Houston, TA, USA

**Keywords:** neural tube, NTDs, organoid

## Abstract

The neural tube is the first critically important structure that develops in the embryo. It serves as the primordium of the central nervous system; therefore, the proper formation of the neural tube is essential to the developing organism. Neural tube defects (NTDs) are severe congenital defects caused by failed neural tube closure during early embryogenesis. The pathogenesis of NTDs is complicated and still not fully understood even after decades of research. While it is an ethically impossible proposition to investigate the in vivo formation process of the neural tube in human embryos, a newly developed technology involving the creation of neural tube organoids serves as an excellent model system with which to study human neural tube formation and the occurrence of NTDs. Herein we reviewed the recent literature on the process of neural tube formation, the progress of NTDs investigations, and particularly the exciting potential to use neural tube organoids to model the cellular and molecular mechanisms underlying the etiology of NTDs.

## 1 | INTRODUCTION

Neural tube defects (NTDs) are one of the most common structural malformations in humans, affecting the development of the central nervous system (CNS). NTDs are considered to be the result of a failure of neural tube closure (NTC) during early embryogenesis. Based on the different locations where abnormal NTC occurs along the anterior-posterior (A-P) axis, NTDs can be divided into many subtypes, including anencephaly, craniorachischisis, and spina bifida. The reported prevalence of NTDs varies from 0.3/10 000 to 199.4/10 000 births around the world.^1^ The estimated prevalence of NTDs in China varies considerably in different regions, ranging from 31.5/10 000 births in northern China^2^ to 5.83/10 000 in southern China.^3^ Despite decades of investigation, the etiology of NTDs in humans has not been clearly elucidated. One of the main reasons for this knowledge gap is the lack of suitable models to study the early developmental events in human embryos.^4^ Fortunately, the newly developed organoids culture system provides a novel three-dimensional (3D) model system with which to study and better understand the development of the human neural tube in an in vitro system. This new model is called the neural tube organoids. In this review, we introduce the formation of the neural tube and the progress of NTDs studies, and particularly the neural tube organoids culture system and its potential applications on NTDs study.

## 2 | NEURAL TUBE DEVELOPMENT AND NTDS

The neural tube is the primordium of the CNS and its proper development is essential to the growth and formation of the embryo. The cells in the neural tube have distinct cell fates programmed in their genome and they differentiate along the body axes. Eventually the anterior part forms the brain, and the remaining portion into the spinal cord. The central cavity in the neural tube becomes the future ventricles and central canal.

The formation of neural tube begins with the differentiation of the ectodermal germ layer. Induced by sonic hedgehog (Shh) signals from the notochord, the overlying ectoderm differentiates into neural ectoderm, and this flat sheet of cells is now referred to as the neural plate.^5,6^ Both sides of the neural plate gradually elevate to form the neural folds. The neural folds rise up and fold toward the midline to complete neural tube closure (NTC).^7^ The bone morphogenetic protein (BMP) and the Wnt signaling pathways also have important functions in the early differentiation of the neural tube.^8–11^ NTC starts in the mid-cervical portion of the embryo, thereby forming the anterior neuropore at the cranial aspect of the embryo and the posterior neuropore at the caudal region. The closure of the anterior neuropore in human occurs around the 25th day post-fertilization, while that of the posterior neuropore closes around day 27. Normally by the end of the fourth week of embryogenesis, NTC is complete.

The neurulation process described above is called primary neurulation, which is one of the two principal modes of neurulation in vertebrates. The other mode is called secondary neurulation.^12^ The major differences are that the neural plate folds and invaginates into the body and separates from the surface ectoderm to form an underlying hollow tube in primary neurulation; while aggregates of mesenchyme cells form a solid cord which undergoes a mesenchymal-epithelial transition (MET) and forms cavities to create a hollow tube in secondary neurulation. Most vertebrates undergo primary neurulation in the anterior region and secondary neurulation in the posterior region around the tail bud. Failure in either primary or secondary neurulation processes could lead to NTDs with different phenotypes.

### 2.1 | The occurrence of NTDs

Failure of proper NTC will cause many open NTDs, including anencephaly, craniorachischisis, meningomyelocele, and myeloschisis among others. In addition, failure of secondary neurulation can lead to closed NTDs, such as spina bifida occulta and myelocystocele.^13^ Both genetic and nongenetic factors play important roles in the etiology of NTDs.

Researchers have identified multiple genetic factors that appear to contribute to the occurrence of human NTDs. For example, our recent study on three independent NTD cohorts (243 cases total) demonstrated that the number of singleton loss-of-function variants is significantly enriched in NTD cohorts compared to that in the controls from the 1000 Genomes Project.^14^ Besides, using the vertebrate animal models, a number of candidate genes were identified that increased the prevalence of NTDs. More than 400 mouse genes have been identified to date, whose abnormal expression is responsible for NTDs.^8,15^ Some of these genes are highly conserved in vertebrates,^16,17^ suggesting a possible relationship of mutations in these genes with potential risk for NTDs in humans. Previous studies have identified that some genetic and chromosomal structural variants are indeed associated with increased risk of NTDs.^18–23^ However, the genotype-phenotype relationship between those genetic variants and NTDs is complicated. Some variants are observed in several different NTD subtypes, while some of these variants have also been identified in healthy individuals.^24^ The functional changes of these variants and how they lead to NTDs remain largely unknown.

In addition to genetic factors, there are multiple nongenetic factors that also contribute to the etiology of NTDs,^25^ including: environmental toxicants,^26,27^ drugs,^28,29^ and maternal factors,^30,31^ such as maternal obesity,^32^ maternal diabetes,^33,34^ and maternal nutritional status especially evidence of a folic acid deficiency,^35,36^ to name just a few. Therefore, the occurrence of NTDs is most likely the result of combinations of complex events, involving both genetic and nongenetic factors.

### 2.2 | Animal models for NTDs

The use of model organisms has always played important roles in the developmental biology research. Using animal models such as the mouse, chick, *Xenopus*, and zebrafish, researchers have successfully constructed many NTD models to study the underlying mechanisms of failed NTC.^37–40^ Many transgenic mouse models have been created to study NTDs, which has been reviewed in many recently published papers.^15,25,41–43^

**FIGURE 1:**
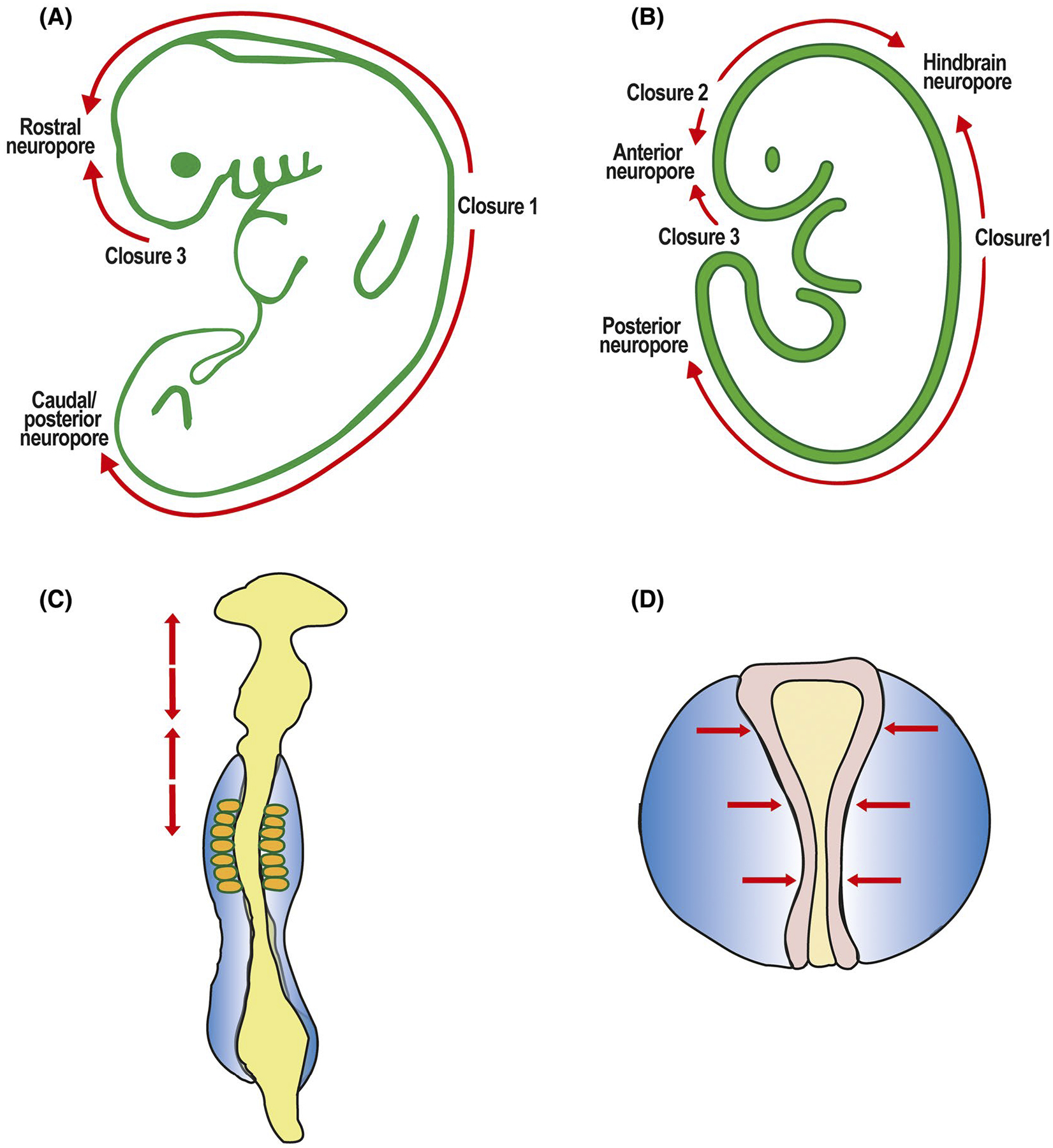
Schematic summary of neural tube closure in different vertebrates. The closure points and directionality are demonstrated for (A) human, (B) mouse, (C) chick, and (D) *Xenopus* embryos, respectively. The arrows indicate directions of closure. The figure displays the neural tubes with the anterior side on the top. For *Xenopus*, neural tube closure starts simultaneously along the entire A-P axis

However, these animal models cannot fully represent human NTDs, as not only are their genomes different from human’s but also the formation of the neural tube are not completely identical among different model species. For example, the process of NTC differs across human, mouse, chicken, and *Xenopus laevis*, especially in the number of initiating closure points, the timing, and sequence of the closing process (Figure 1). Although NTC in mouse embryos closely resembles that of human, there are still some differences between mouse and human NTC. The mouse has three initiating closure points while humans have only two initiating closure points (Figure 1A,B). The initiating closure points in human embryos are equivalent to the closure point 1 and 3 in the mouse.^44^ As illustrated in Figure 1B, mouse closure point 1 is located at the hindbrain/cervical boundary while closure point 2 is located at the forebrain/midbrain boundary. Both closure point 1 and 2 are bi-directional. The closure point 3 is located at the anterior-most end of the neural tube and only closes in a posterior direction. The zippering process of NTC initiates from the closure study point 1 and 2, and then expands along both directions of the closure points. At the same time, the closure point 3 proceeds caudally from this site toward closure point 2.^8^ Chicken embryos also have only two initial closure points, which are located at the future midbrain and at the hindbrain/cervical boundary, respectively, and both of these sites undergo a bi-directional closure process^12^ (Figure 1C). The neural tube closure in *Xenopus laevis* is quite different as it occurs almost simultaneously along the entire body axis^45^ (Figure 1D).

Therefore, vertebrate animal models have limitations in fully replicating the precise developmental events of the human neural tube in vivo, as well as the etiology of human NTDs. However, it is impossible to perform human neural tube development research in vivo due to ethical considerations. Fortunately, with the extensive application of an emerging new technology-organoid culturing, researchers can now build 3D cell models of the neural tube from human pluripotent stem cells (hPSCs). This 3D cell model is referred to as neural tube organoids. The cell aggregates in neural tube organoids simulate the in vivo cell composition of the neural tube. This new model system makes it possible to study the human neural tube development utilizing an in vitro culture system. We will summarize this new approach and its application in NTD research in the following sections.

## 3 | NEURAL TUBE ORGANOIDS CULTURE

Organoids are defined as 3D cell aggregates generated by pluripotent stem cells or organ progenitors, consisting of organ-specific cell types that self-organize through cell sorting and spatially restricted linage commitment similar to in vivo.^46,47^ Organoids can be derived from embryonic stem cells (ESCs), adult stem cells (ASCs), and induced pluripotent stem cells (iPSCs). Under certain extracellular scaffolding culture conditions and signaling factors, these cells are induced to rearrange through self-organization mechanism to form proper cell aggregates. Organoids can represent both the phenotypic and genetic characteristics of corresponding organs and are able to sustain these characteristics during the whole in vitro culturing process. Most importantly, two key events in organogenesis are faithfully reproduced during the formation of organoids, which are cell sorting and spatially restricted lineage commitment.^46^ These properties make organoids a more suitable model to simulate not only the in vivo organogenesis process, but also the physiological or pathological states of organs and tissues. In order to study the functions of many different human organs in vitro, organoids corresponding to different organs have been successfully constructed in culture systems, including the cerebrum,^48^ retina,^49^ heart,^50^ liver,^51^ kidney,^52^ stomach,^53^ and intestine.^54^ As organoids can be generated from individual iPSCs, organoids have been adapted to clinical studies.^55,56^

Compared to some other organs, neural tube organoids have been developed relatively late even though the neural tube is a fairly simple organ. It was developed after the creation of cerebral organoids.^48^ In general, neural tube organoid culture is based on the self-organization ability of PSCs under 3D extracellular matrix conditions and exogenous signaling factors delivered at the appropriate time point to activate the corresponding signaling pathways. Scientists have successfully generated neural tube organoids from both human and mouse PSCs.

### 3.1 | Culturing methods

**FIGURE 2:**
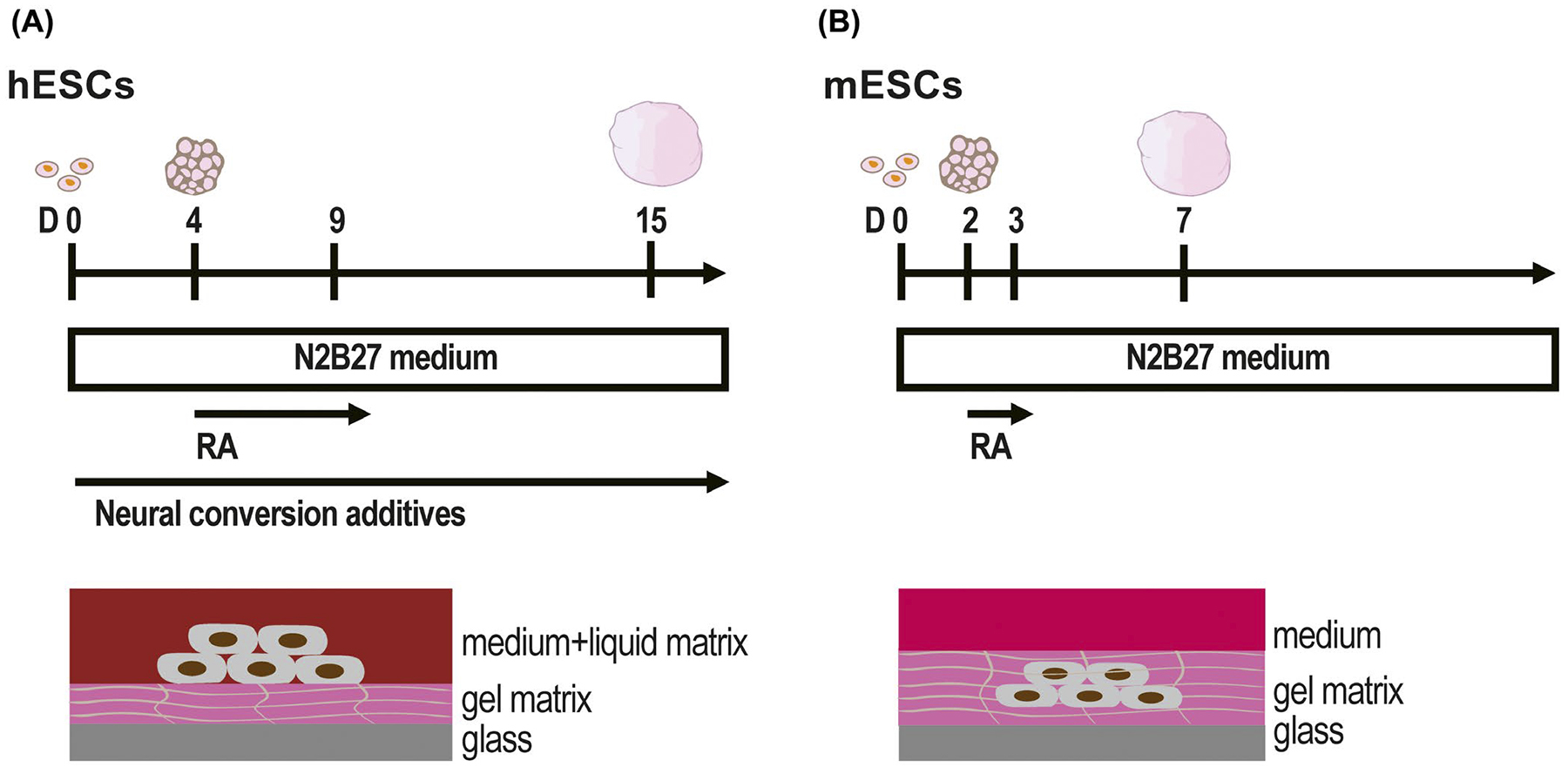
Diagrams for neural tube organoid culture medium and methods from either human (A) or mouse PSCs (B). Culturing medium and additives (RA and other neural conversion additives) were applied at different time period. Neural conversion additives could be added to the culture for human organoids. The bottom panels briefly illustrated the methods for human and mouse organoids, noticeably the mouse ESCs are normally embedded in the matrix while hESCs are placed on gel bed in the Gel-3D method^63^

To form a proper neural tube organoid, the culture methods are very important and must be rigidly controlled. We summarized the development of the methods for neural tube organoids in Table 1. Although the first neural tube organoid^57^ was cultured after the development of cerebral organoids,^48^ a similar idea can be traced back to 2011, when Sharon and colleagues cultured suspended embryonic bodies (EBs) to study the gastrula-organizer.^58^ In 2014, Meinhardt and co-workers successfully cultured the first neural tube organoid using mouse ESCs in Matrigel.^57^ Two years later, Ranga et al used similar methods to study how the 3D microenvironment influences the neural tube organoids’ D-V pattern formation.^59^ They added bFGF in their culture system to enhance the neuromesodermal development. Subsequently, several papers have been published building “spinal organoids” using hPSCs either embedded in Matrigel^60^ or suspended medium to induce EB-like structure.^61,62^ Scientists also added more additives to induce certain cell fates, such as spinal motor neuron^60^ or D-V patterned spinal cord-like tissues.^61^ In past 2 years, more advances with respect to the culture methods have been developed for neural tube organoids. Zheng and colleagues developed a Gel-3D method in which 2% liquid form of Geltrex was added to the induction medium to create a 3D extracellular matrix (ECM) environment for the hESCs to grow into neural tube organoids^63^ (also illustrated in Figure 2). This method could generate patterned neural rosettes. Veenvliet and co-workers cultured organoids with “trunk-like structure (TLS),” which mimic the posterior neural tube and bilateral somites.^64^ They let mESCs aggregates to form gastruloids first and then, embedded these gastruloids into 5% Matrigel to further support organoid development. Interestingly, the neural tube organoids started from *Tbx6*^−/−^ mESCs also recaptured some in vivo phenotype at the molecular level.

As the culturing matrix and medium are crucial for neural tube organoid culture, we continue to discuss these two parts in the following sections.

#### 3.1.1 | Culturing matrix

The culturing matrix is used to support the 3D growth of the organoids and increase cell-cell and cell-matrix interactions so as to promote ESCs to form functional and organ-like structures.^65^ As the growth of organoids is dependent on not only the biomaterials but also on the environment, many aspects of the culturing matrix can influence the outcome of organoid culture. These aspects include whether culturing matrix is used or not, how the culturing matrix is used, and the properties of the culturing matrix that is used in the system. Currently there are three major types of culturing matrices including: (a) the basement membrane extract from organisms (eg, Matrigel, Geltrex), (b) the purified basement membrane extract (eg, laminin/entactin complex gel), and (c) the synthetic matrix (eg, PEG-based gel). For example, Zheng and co-workers used Geltrex^63^; Ranga and colleagues studied the PEG-based gel with different components^59^; and Meinhardt and co-workers successfully applied all three types of culturing matrixes in their study.^57^ There are two major methods dealing with how to apply the culturing matrix. One method is to mix the starting cells with culturing matrix in a liquid state and then, allow the mix to solidify. Using this approach, the cells are embedded in the culturing matrix after solidification. In the other method, starting cells are seeded directly on the surface of a solid culturing matrix and the liquid culturing medium mixed with liquid matrix are added to cover the cells.

#### 3.1.2 | Culturing medium

The basic culturing medium for neural tube organoids is N2B27 medium. N2B27 medium is named after the N2 and B27 supplements. Its basic constituents are DMEM/F12 and neurobasal medium in a volume ratio at 1:1, and supplied with B27 supplements, N2 supplements, β-mercaptoethanol, and with additional growth factors that vary based on unique applications. DMEM/F12 medium is a basal medium that can sustain many different mammalian cell types. Neurobasal medium and B27 supplements are used to maintain the growth and maturation of embryonic neuronal cells, without the need of an astrocyte feeder layer. N2 supplements can sustain neurons. Finally, β-mercaptoethanol is used to control the redox environment of the culture.

The neural tube organoids cultured with N2B27 medium can form a basic lumen structure. However, its structure and cell compositions remain far less complicated than a neural tube in vivo. Therefore, exogenous signals are added to generate the correct pattern formation of organoids. The most often used additives are retinoic acid (RA) and Shh to achieve proper D-V patterning^57,63^ (Figure 2). Other additives are added to improve growth and induce patterns in the organoids. For example, Wnt signaling is required for the differentiation of A-P pattern.^66^ Therefore, the application of GSK3i, an activator of the Wnt signaling pathway, can enhance the posterior differentiation in the neural tube organoids.^67^ Furthermore, applying several different signaling molecules together can induce neural tube organoids to mimic-specific region of the neural tube, such that the GSK3i-SHH combination can induce neural tube organoids with forebrain-specific pattern (NKX2.1^+^/SIX6^+^).^67^

### 3.2 | Comparison with neural tube in vivo development

Organoids were established to simulate in vivo organs or tissues. Previously, the similarities between neural tube organoids and in vivo neural tube were analyzed based on the following features: overall structure, neural fate commitment, apical-basal polarity, A-P and D-V patterns.

The first and easiest way to assess the characteristics of the neural tube organoids is to observe the overall structure directly under a light microscope. Most neural tube organoids reported in the literature have a cavity similar to that of neural tubes developing in vivo. The cavities of organoids were cystic shaped as the organoids are nearly globular structures, while the neural tube forms a long columnar shape cavity in vivo.

Other organoid features are observed by immunofluorescent staining with specific markers, which will be discussed in detail below. Similar to the in vivo developmental process, many features are time-dependent. Therefore, temporal information is often emphasized when describing a feature of neural tube organoids.

#### 3.2.1 | Cell fate commitment and axis patterns

Similar to in vivo embryonic development, cell fate is also changed during neural tube organoid formation. In each stage, characteristic proteins were expressed and can be used as markers. OCT4, NANOG, and SSEA4 are expressed in pluripotent cells; while NESTIN, MUSASHI, SOX1, SOX2, and PAX6 can be detected in neural stem cells. Sometimes the expression of neuronal makers can also be examined, like βIII-TUBULIN, MAP2, and NeuN.

**FIGURE 3:**
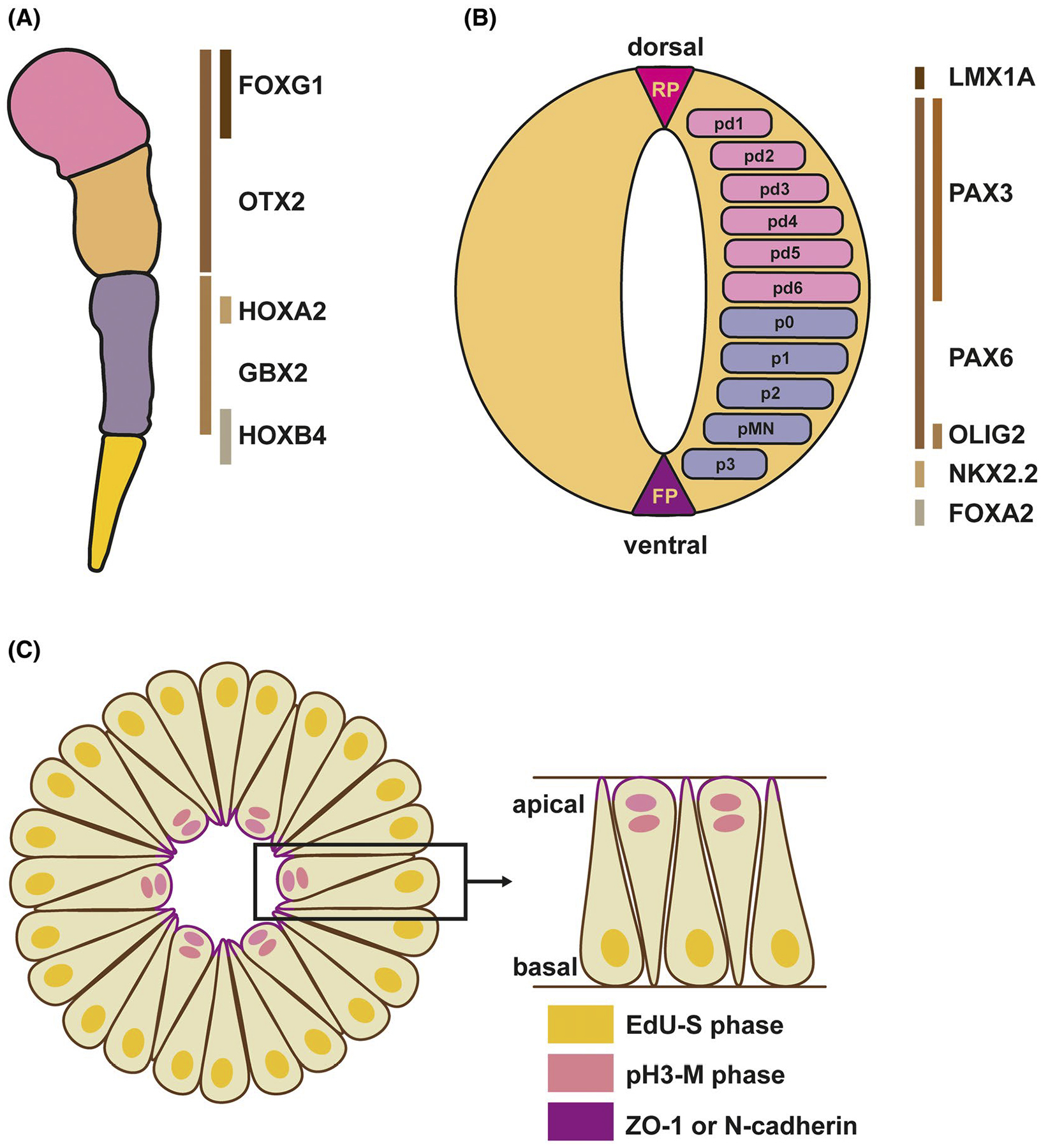
A-P and D-V axis markers used in characterizing neural tube organoids, and apical polarity and IKNM in neural tube organoids. Different markers are expressed at different regions of embryos along the A-P (A) and D-V (B) axis. (C) The apical polarity markers, such as ZO-1 and N-cadherin, are located inside the neural rosette, which is the apical side. The IKNM can be observed by nuclei position, nuclei at S phase (EdU positive) are at the basal side and nuclei at M phases (pH3 positive). RP, roof plate. FP, floor plate. IKNM, interkinetic nuclear migration

In addition to cell fate markers, position-specific markers are often used, especially along the A-P or D-V axis. For the A-P axis, marker proteins for human embryos include: FOXG1 (forebrain), OTX2 (forebrain and midbrain), HOXA2 (r2 of hindbrain), GBX2 (hindbrain), HOXB4 (cervical spinal cord, anterior border at r6/r7 boundary of hindbrain),^63^ to name just a few (Figure 3A). To date, most neural tube organoids can mimic only a small/local region along the A-P axis instead of the entire neural tube from head to tail. For example, Zheng and colleagues cultured neural tube organoids with an identity of the posterior hindbrain and the cervical spinal cord.^63^ Such limitations may be over-come by applying organoid-on-chip method, a micro-fabricated cell culture device to generate organoids in a more reproducible manner.^68^ This kind of device is constructed by a removable platform with a central chamber for matrix loading and organoids culturing, and lateral fluid-through channels that supply medium and growth factors. Compared to traditional culture methods, microfluidics control the culture environment more accurately and thus enable the culture environment to more closely resemble the in vivo situation. Recently, Rifes and co-workers cultured hESCs layer on Matrigel in a chip and successfully generated a neural tissue with the A-P pattern of the embryonic brain.^67^ Although it is a multicellular tissue rather than a tube-like structure, this is the first attempt to achieve a longer range A-P patterned multicellular tissue.

For the D-V axis, previous studies reported that progenitor cells located close to the neural tube lumen could be divided into pd1–6, p0–2, pMN, and p3, totaling 11 regions along the D-V axis (Figure 3B). Neurons located further away from the lumen could also be divided into 11 regions along the D-V axis. Each region expresses specific transcription factors, which can also be used to characterize the D-V pattern in the neural tube organoids. For example, PAX3 is expressed in the dorsal pd1–6 regions^69,70^; PAX6 is expressed in the pd1–6, p0–2, and pMN regions; OLIG2 is expressed in the pMN region; NKX2.2 is expressed in the p3 region. And for neurons, ISL1/2/HB9 are expressed in motor neurons. In addition, LMX1A is expressed in the roof plate and FOXA2/ARX/SHH are expressed in the floor plate.^71^ Some of the established neural tube organoids achieved the proper D-V pattern to some extent. For example, a PAX3-OLIG2-NKX2.2-FOXA2 patterned organoid was established by Zheng et al.^63^

In order to ensure proper formation of neural tube organoids, proteins marking cell linages outside of the neural tube are often used to show that these cells do not appear in the neural tube organoids. Some of the markers used in previous studies include: E-CADHERIN for presumptive epidermis, SOX10 for neural crest, SOX17 for endosperm, BRACHYURY/CDX2/EOMES for primitive streak and mesoderm.

Apart from these traditional methods, recently single-cell sequencing method also revealed neural tube organoids shared conserved transcriptome patterns with neural tubes in vivo. Veenvliet and colleagues performed single-cell sequencing on their TLS organoids, and found that these organoids follow the same stepwise gene regulatory programs as the mouse embryo.^64^

#### 3.2.2 | Apical-basal polarity

The cells in neural tube have very typical apical-basal polarity. ZO-1 and N-cadherin are often used to label the cellular polarity. ZO-1 is a membrane protein located at tight junctions. N-cadherin is a transmembrane cell adhesion protein. Both proteins located at the apical side of cells. Most of the previously described neural tube organoids had polarity comparable to that of neural tubes that are developing in vivo, in which their apical sides are facing the inside lumen of the organoids.

Only one study had reported a neural tube organoid with inverted polarity as its apical side was facing the outer surface rather than the inner surface of the cavity.^61^ The authors of this study used suspended culture instead of a solid matrix. It has been reported that adhesion ligands, or more importantly, 3D culture environments might be crucial for apical-basal polarity in epithelial tissues.^72^ So, it is possible that lacking adhesion ligands in the suspension culture could explain the observed inverted polarity.

It has been reported that cell nuclei in the neural tube have very characteristic movements during mitosis in vivo. They are close to the basal surface during S phase, and close to the apical surface during M phase. This phenomenon is called interkinetic nuclear migration (IKNM). Whether IKNM also occurs in neural tube organoids could be examined by observing the nuclei at S and M phases. EdU, a thymidine analog, is usually used to label nuclei at S phase, and phosphorylated histone H3 is often used to label nuclei at M phase (Figure 3C). When the neural tube organoids develop correct apical polarity, they also have correct IKNM similar to the in vivo process.^57,63^

## 4 | POTENTIAL DEVELOPMENT AND APPLICATIONS OF NEURAL TUBE ORGANOIDS

In this review we have briefly introduced the development of neural tube and how NTDs occur. We have also summarized current methods for neural tube organoid culture, which can be used as an in vitro model for studying human NTDs.

Clearly the culture conditions are extremely important for organoid growth. In the future, the culture conditions of neural tube organoids can be further optimized to more closely mimic the in vivo development processes of the in vivo neural tube, for examples, developing a morphologically closing process, and fully developed AP, DV patterns. Improvements in several aspects of organoid biology can be made. (a) Optimizing culturing medium. Currently, some studies have been published using different culturing mediums with or without additions of morphogens and other molecules. Most molecules were involved in critical signaling pathways in development, including Wnt,^59,60,63^ Shh,^57,59,61,63^ BMP,^59,61^ Notch,^59^ FGF,^59^ RhoA/Rho Kinase signaling pathways,^59^ and so on. In the future, a broader spectrum of additives could be considered with more precise control of concentrations, starting point, time period applied to the organoid culture system, and different combinations of additives. Apart from the additives, basic nutrient contents could also be studied to achieve a better control of the metabolism, and thus the state of the culture. The organoid-on-chip method may be helpful on this point. (b) Optimizing the culture matrix. The matrix components can affect the growth and development of organoids. Therefore, components including different extracellular matrices with different matrix metalloproteinase sensitivity and physical properties can be considered.^59^ (c) Finer control of culture environment. Utilizing technologies like microfluidics could provide finer control on the application of different morphogens and growth factors with respect to both the timing and the location of their application. However, other factors may also need to be considered. For example, a recent study has shown that mechanical force of culture environment can also affect neural tube organoids growth and patterning.^73^ All these three improvements will make neural tube organoids more reliable and more similar to the in vivo neural tube. However, one limitation of current neural tube organoids is that it cannot mimic the NTC process even though they have a lumen structure. Determining how to induce and mimic the NTC process in neural tube organoids will be the biggest challenge in the future.

Nonetheless, neural tube organoids can still serve as an excellent model to study the pathological processes of NTDs. Compared to animal models, it would be easy to apply exogenous pathogenic factors including suspected environmental toxicants into the organoid culture medium or matrix to study their impact. This would facilitate evaluating the dosage and toxic effects of a wide range of extracellular pathogenic factors. Second, intracellular pathogenic factors could be studied by interfering the starting cells. Not only patient-derived hiPSCs can be used as starting cells, but also other previously reported genetic variants can be imported into the starting cells through genome editing. That is, hPSCs can be edited by CRISPR/Cas9 methods to contain the genetic variants that have previously been reported to be associated with NTDs. The edited hPSCs can be used as the starting cells for culturing neural tube organoids. By comparing these organoids to the organoids initiated from wild-type hPSCs, we can have better understanding of the functional changes of the NTD- associated genetic variants. Furthermore, it is also easier to test drugs/nutrients treatment for NTDs using this approach. Taken together, neural tube organoids are excellent in vitro models for studying neural tube development, pathogenesis, and management of the comorbidities associated with NTDs.

## Figures and Tables

**TABLE 1:** The development of neural tube organoids

Year	Reference	Starting cells and method	Culture purpose
2011	Sharon et al^58^	hESCs in medium	Differentiated EBs as the Gastrula-organizer
2013	Lancaster et al^48^	hESCs-derived EBs in Matrigel	Cerebral organoids
2014	Meinhardt et al^57^	mESCs in Matrigel	Neural tube organoids: patterned neural tube
2016	Ranga et al^59^	mESCs in Matrigel or hydrogel	Neural tube organoids: Neuroepithelial cysts with D-V pattern
2016	Demers et al^60^	hESCs in Matrigel, a 3D-device	Spinal organoids: spinal motor neuron differentiation
2018	Ogura et al^61^	hiPSCs, suspended in medium	Spinal organoids with D-V pattern
2019	Duval et al^62^	hESCs or mESCs, suspended in medium	Spinal organoids with D-V pattern
2019	Zheng et al^63^	hESCs on gel bed + 3D ECM, which is 2% Geltrex with medium (Gel-3D)	Neural tube organoids: patterned neural rosettes
2020	Veenvliet et al^64^	mESCs, 96-hour gastruloids embedded in 5% Matrigel	Neural tube organoids with trunk-like structure

## Abbreviations:

3D: three-dimensional
A-P axis: anterior-posterior axis
ASCs: adult stem cells
bFGF: basic fibroblast growth factor
BMP: bone morphogenetic protein
CNS: central nervous system
D-V pattern: dorsal-ventral pattern
ESCs: embryonic stem cells
hPSCs: human pluripotent stem cells
IKNM: interkinetic nuclear migration
iPSCs: induced pluripotent stem cells
NTC: neural tube closure
NTDs: neural tube defects
RA: retinoic acid
Shh: sonic hedgehog
